# Supplementary material for: Taxonomic revision of the tarantula genus Aphonopelma Pocock, 1901 (Araneae, Mygalomorphae, Theraphosidae) within the United States
Source: Zookeys. 2016 Feb 4;(560):1–340. doi: 10.3897/zookeys.560.6264 (PMC4768370; doi:10.3897/zookeys.560.6264)
Supplement: Supplementary material 2 — Compressed files (.zip) containing Aphonopelma boxplot and PCA morphospace data. [file zookeys-560-001-s002.zip › Supp2b.zip!/Appendix 2B/Boxplot_morphospace/Aphonopelma_boxplots_males/ALL/P4M4_ratio_males.pdf]

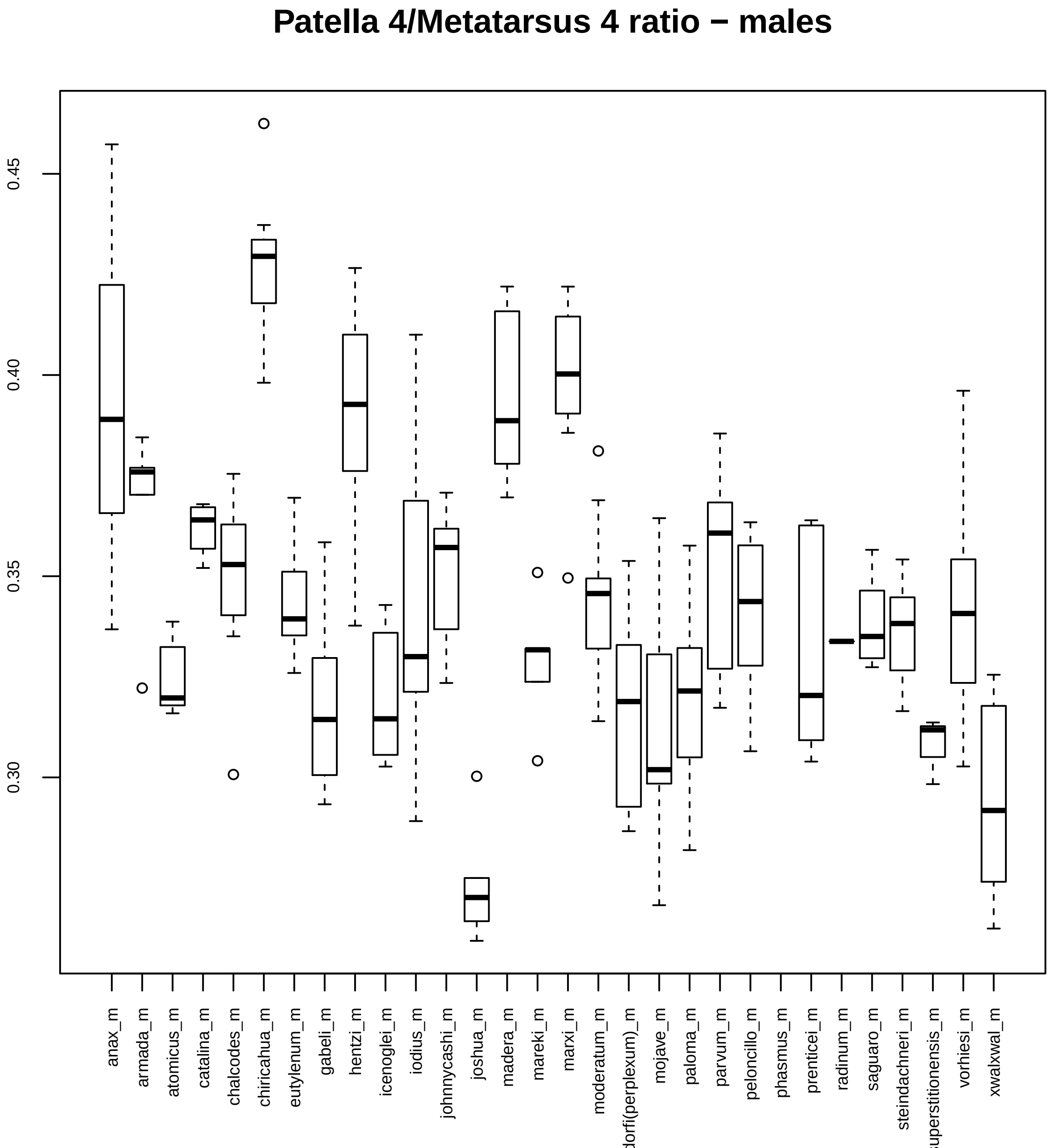
Patella 4/Metatarsus 4 ratio − males
0.30
0.35
0.40
0.45
anax_m
armada_m
atomicus_m
catalina_m
chalcodes_m
chiricahua_m
eutylenum_m
gabeli_m
hentzi_m
icenoglei_m
iodius_m
johnnycashi_m
joshua_m
madera_m
mareki_m
marxi_m
moderatum_m
mojave_m
paloma_m
parvum_m
peloncillo_m
phasmus_m
prenticei_m
radinum_m
saguaro_m
steindachneri_m
vorhiesi_m
xwalxwal_m
